# Psychophysiological and emotional effects of human–Dog interactions by activity type: An electroencephalogram study

**DOI:** 10.1371/journal.pone.0298384

**Published:** 2024-03-13

**Authors:** Onyoo Yoo, YuTong Wu, Jin Soo Han, Sin-Ae Park

**Affiliations:** 1 Department of Bio and Healing Convergence, Graduate School, Konkuk University, Seoul, South Korea; 2 Department of Laboratory Animal Medicine, Institute for the 3Rs & Animal Welfare, College of Veterinary Medicine, Konkuk University, Seoul, South Korea; 3 Department of Systems Biotechnology, Konkuk University, Seoul, South Korea; Belgrade University Faculty of Medicine, SERBIA

## Abstract

Animal-assisted interventions are being increasingly used in studies that support various health effects. This study compared the psychophysiological and emotional responses during diverse activities with a dog to understand the impact of activity type. This study included 30 healthy adults (average age: 27.9 ± 8.4 years). Participants performed eight different activities with a dog for 3 minutes each. These activities included meeting, playing, feeding, massaging, grooming, photographing, hugging, and walking. Brain waves in the prefrontal, frontal, parietal, and occipital lobes were measured during the activities. Subjective evaluation of their emotions was recorded after each activity via the Profile of Mood States, Semantic Differential Method, and Stress Numeric Rating Scale. The alpha (relative, relative slow, relative fast) power spectra indicated that the brain’s relaxation and resting state significantly increased when playing with and walking a dog. The beta (relative, relative low, and relative mid) power spectra significantly increased during dog massage, grooming, and playing activities, indicating improved concentration without stress. Notably, playing with a dog positively affected both relaxation and concentration. The Profile of Mood States outcome showed that activities such as feeding, massaging, and hugging the dog decreased the total mood disorder score, which indicated a positive effect on participants’ moods. The Semantic Differential Method revealed that participants felt comfortable and natural while walking with a dog and relaxed when massaging it. Participants showed significantly lower stress moods in all the activities. This study demonstrated that specific dog activities could activate stronger relaxation, emotional stability, attention, concentration, and creativity by facilitating increased brain activity. In addition, interactions with dogs could decrease stress and induce positive emotional responses. These results provide data that forms the basis for the composition of the AAI program and may be applicable as a reference to determine the most effective activities for specific applications.

## 1. Introduction

Dogs were domesticated more than 30,000 years ago [1], and have assisted humans in numerous tasks, including hunting, working, herding, and guarding throughout history. Dogs can communicate with people [2]. They have been faithful friends to humans and share emotions beyond that of an efficient assistant.

Several studies have reported the physiological and emotional benefits of interactions with animals, especially dogs. Interaction with dogs increases oxytocin concentrations [3, 4], decreases cortisol levels [4, 5], and reduces the risk of cardiovascular disease [6]. Interaction with animals also reduces stress reactivity, anxiety, and behavioral distress and is considered an effective treatment for mental and behavioral disorders [7–9]. Owing to these health benefits, animal-assisted interventions (AAI) are being increasingly used in diverse fields. AAI, as defined by the International Association of Human-Animal Interaction Organizations [10], is a “goal oriented and structured intervention that intentionally includes or incorporates animals in health, education, and human services (e.g., social work) for the purpose of therapeutic gains in humans.” It encompasses animal-assisted therapy (AAT) provided by professionals in health, education, or human services; animal-assisted education (AAE) carried out by educational and related service professionals; and animal-assisted activity (AAA), involving informal interactions and visitations by the human-animal team for motivational, educational, and recreational purposes.

Although various health effects of interactions with animals have been reported, most studies are based on a holistic approach and compared health effects before and after or between experimental and control groups. Studies on human–animal interaction effects by activity type are scarce. A recent systematic literature review based on data from 129 studies on human–animal interactions reported that further in-depth studies are required to identify the benefits of activity types [11]. Additionally, research on brain activity mechanisms that correlate to human–animal interaction effects is incipient and insufficient.

The information received and processed by the body triggers diverse physiological responses, which are reflected in distinct brainwave patterns [12]. The EEG technique is a valuable tool for investigating the psychological processes associated with human perception and behavior [13]. It offers precise and immediate information, enabling the detection of unconscious and swift processes that may not be revealed through self-disclosure [13]. This study aims to investigate the effect of interactions with a dog on different activity types by measuring psychophysiological responses via an electroencephalogram (EEG) and assessing emotional responses using subjective mood questionnaires.

## 2. Materials and methods

### 2.1. Participants

Participants were recruited between May and June 2022. Recruitment notices were posted in pet salons and a dog beauty academy in Seongnam, South Korea. In total, 30 adults in their 20s to 40s (15 men, 15 women; average age: 27.9 ± 8.4 years) participated. Individuals without allergies or cynophobia were selected. Exclusion criteria were participants with a history of cardiovascular diseases, such as high blood pressure, unstable angina, heart attack, heart surgery, psychopathological diseases, who took related drugs, or who were pregnant or lactating. Participants were asked not to smoke or drink caffeinated beverages within three hours before the activity to avoid potential stimulation effects [14]. Before the experiment began, participants were informed of the study contents and precautions, and written informed consent was obtained. Participants were also asked to complete a demographic questionnaire that included questions on age, sex, height, and weight. An incentive (a product worth KRW 15,000 per time) was provided to those who participated. This study was approved by the Institutional Bioethics Committee of Konkuk University (7001355-202201-HR-504). Collected data were recorded and maintained in a numbered manner, and the authors had no access to information that could identify individual participants after data collection.

### 2.2. Experimental environment

The experiment was conducted in an independent office space (9.7m x 3.7m) at Bellaluci Grooming Academy, located in Seongnam, South Korea. The space was sufficient to proceed with the activity and a handler was on standby. It had a white ceiling and walls without any decoration, was quiet, and blocked external noise to reduce the potential influence. The room condition was regulated consistently: average temperature, 23.2 ± 0.4°C, humidity, 55.8 ± 4.8%, and illuminance, 645.9 ± 87.8 lx.

The ending activity (walk) was conducted on a park trail located at a three-minute walking distance. The outdoor weather conditions were constant in May, and all the schedules were conducted during the daytime in fine weather. We considered places familiar to the participating dog; that is, the experiment settings (office and park trail) selected were where the dog spent time daily.

### 2.3. Dog

A four-year-old female Standard Poodle participated. The dog was ready for this type of work had a compatible personality, and was fully trained in basic obedience, manners, aggression, and sociability as verified through its prior participation in numerous dog shows. The dog was registered with the Korean Kennel Club (PS-B80005) and qualified as an AAA dog after passing the Korean Kennel Club certification evaluation. The dog was vaccinated, and veterinary examinations were performed regularly during health checkups. She was thoroughly managed to ensure that there were no diseases or parasites and was not fed raw meat or other unprocessed raw protein. She was bathed and groomed periodically before and during the study period. A professional dog handler was on standby to control and protect the dog.

After consultation with the professional dog handler, to avoid overworking the dog, a schedule of approximately three times a day was deemed appropriate, considering the dog’s daily average exercise amount and health condition. Accordingly, the session was limited to a maximum of three times a day with an activity time of approximately 60 minutes per session. The dog was owned by the lead researcher, and consent was obtained for all activities. This study did not include any invasive intervention or drug treatment for the dog and, therefore, did not require approval from the Institutional Animal Care and Use Committee, South Korea. All sessions were conducted according to the guidelines of the International Association for Human-Animal Interaction Organizations [10].

### 2.4. Experimental protocol

**Fig 1:**
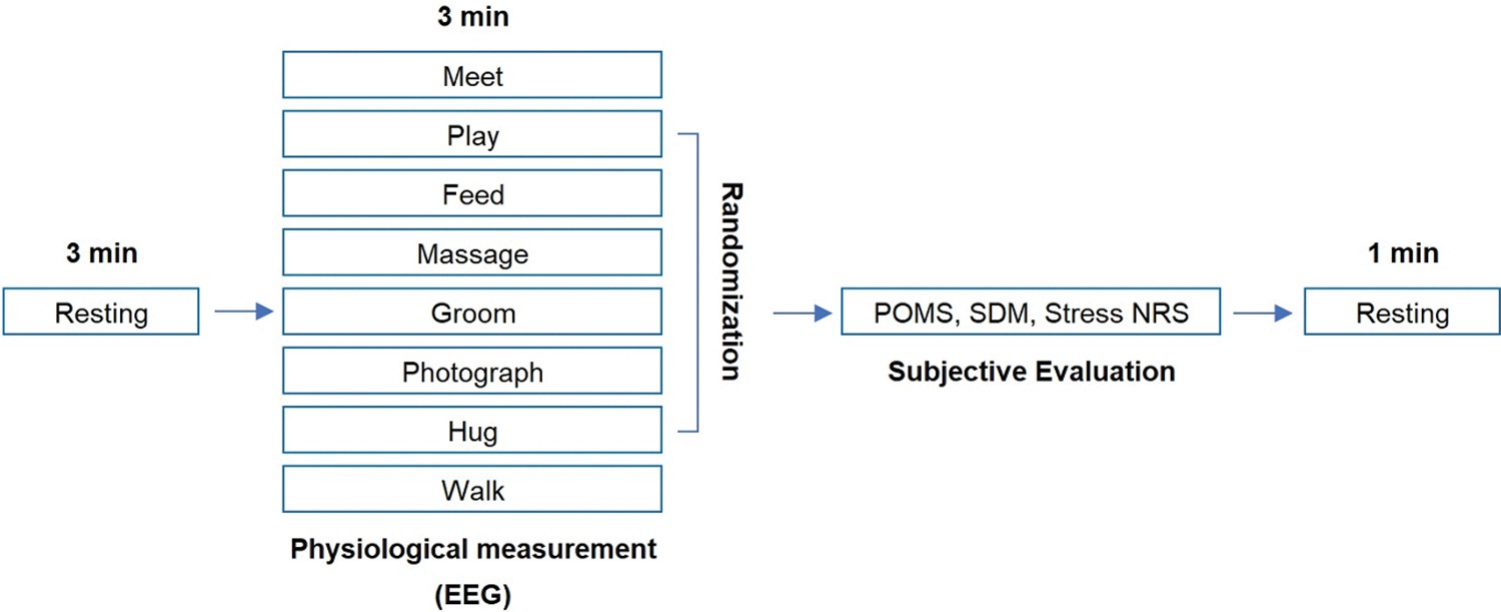
Study procedure. EEG = electroencephalography; POMS = Profile of Mood State; SDM = Semantic Differential Method; Stress NRS = Stress Numeric Rating Scale.

**Fig 2:**
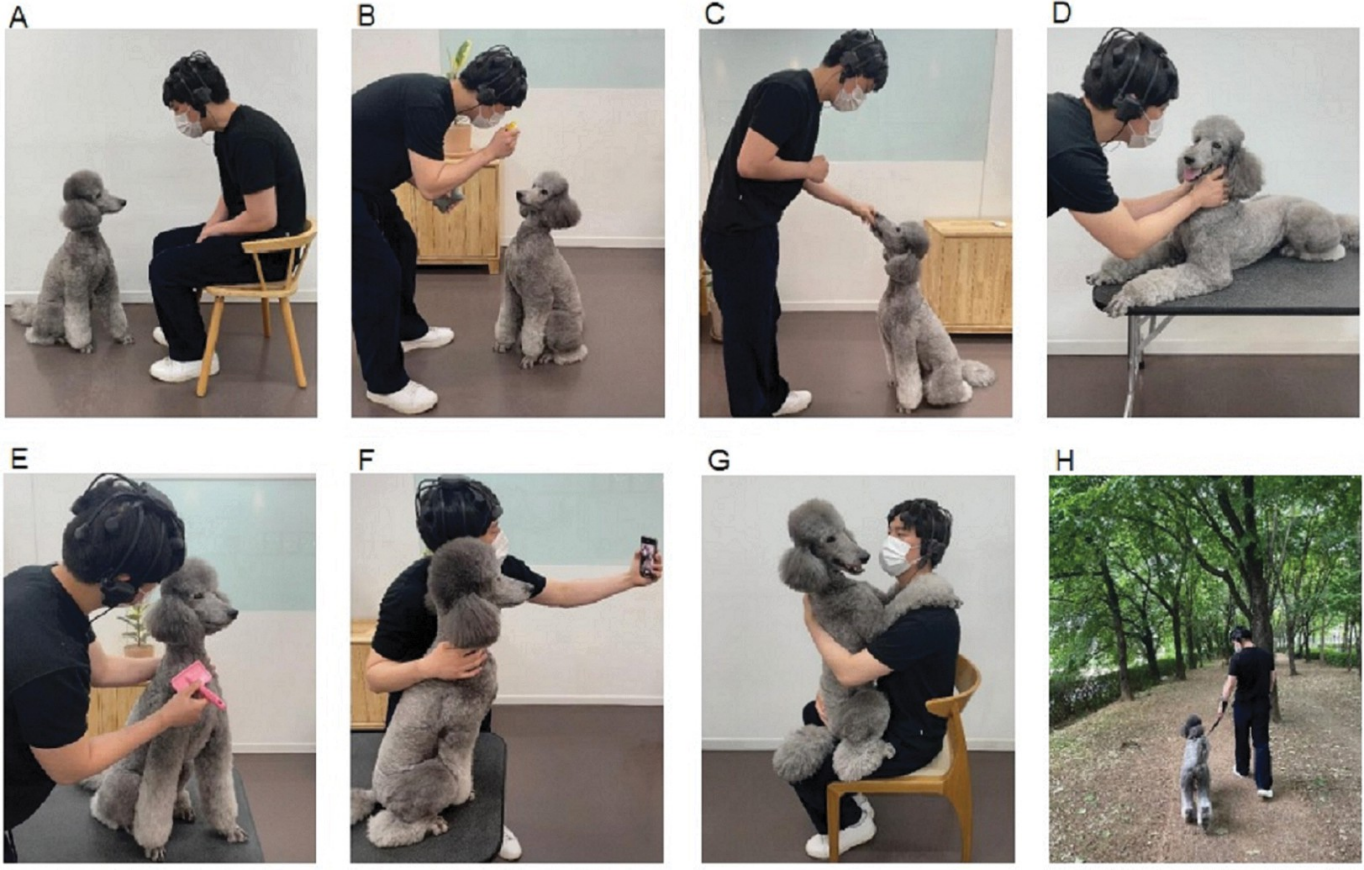
The appearance of animal-assisted activities performed by the participants. (A) meet, (B) play, (C) feed, (D) massage, (E) groom, (F) photograph, (G) hug, and (H) walk.

Participants performed the study procedure shown in Fig 1. In total, eight AAAs were conducted for each participant in a single session: meeting, play, feeding, massage, grooming, photography, hugging, and walking (Table 1 and Fig 2). These activities were primarily selected to prioritize direct interactions with the dog, encompassing regular activities that people typically engage in with their canine companions, rather than incorporating animals as assistants. Before the activity began, the participants had 3 minutes of rest by sitting in a chair and staring at a wall to minimize stimulation. Considering the dog’s mood to adapt to the stranger, the beginning and ending activities were fixed (meeting and walking, respectively), whereas the other activities were performed in random order. Brain waves were measured for 3 minutes per stimulus and participants were instructed not to speak or make rushed movements. A detailed demonstration of each activity was briefed before the experiment.

**Table 1:** Description of animal-assisted activities performed by the participants.

Activity	Description
Meet	Make eye contact and observe the dog
Play	Play with hand-sized squeaking toys
Feed	Feed snack (5–6 small pieces of dog treats)
Massage	Give the dog a gentle massage
Groom	Softly brush the dog with a hand-sized slicker brush of 30g
Photograph	Take a picture of the dog or together with the dog
Hug	Carefully hug and feel the heartbeat of the dog
Walk	Stroll the nearby park trails with the dog

Participants had 1–2 minutes to complete a questionnaire immediately after each activity, reporting their subjective emotional states via the Profile of Mood State (POMS), Semantic Differential Method (SDM), and Stress Numeric Rating Scale (Stress NRS). And took a 1-minute rest before starting the next activity. The experiment concluded after all eight activities were completed. All the procedures, including a total of eight activities, were completed within 60 minutes. No unintended or unfavorable situations occurred during data collection.

### 2.5. Measurements

#### 2.5.1 Electroencephalogram (EEG)

**Fig 3:**
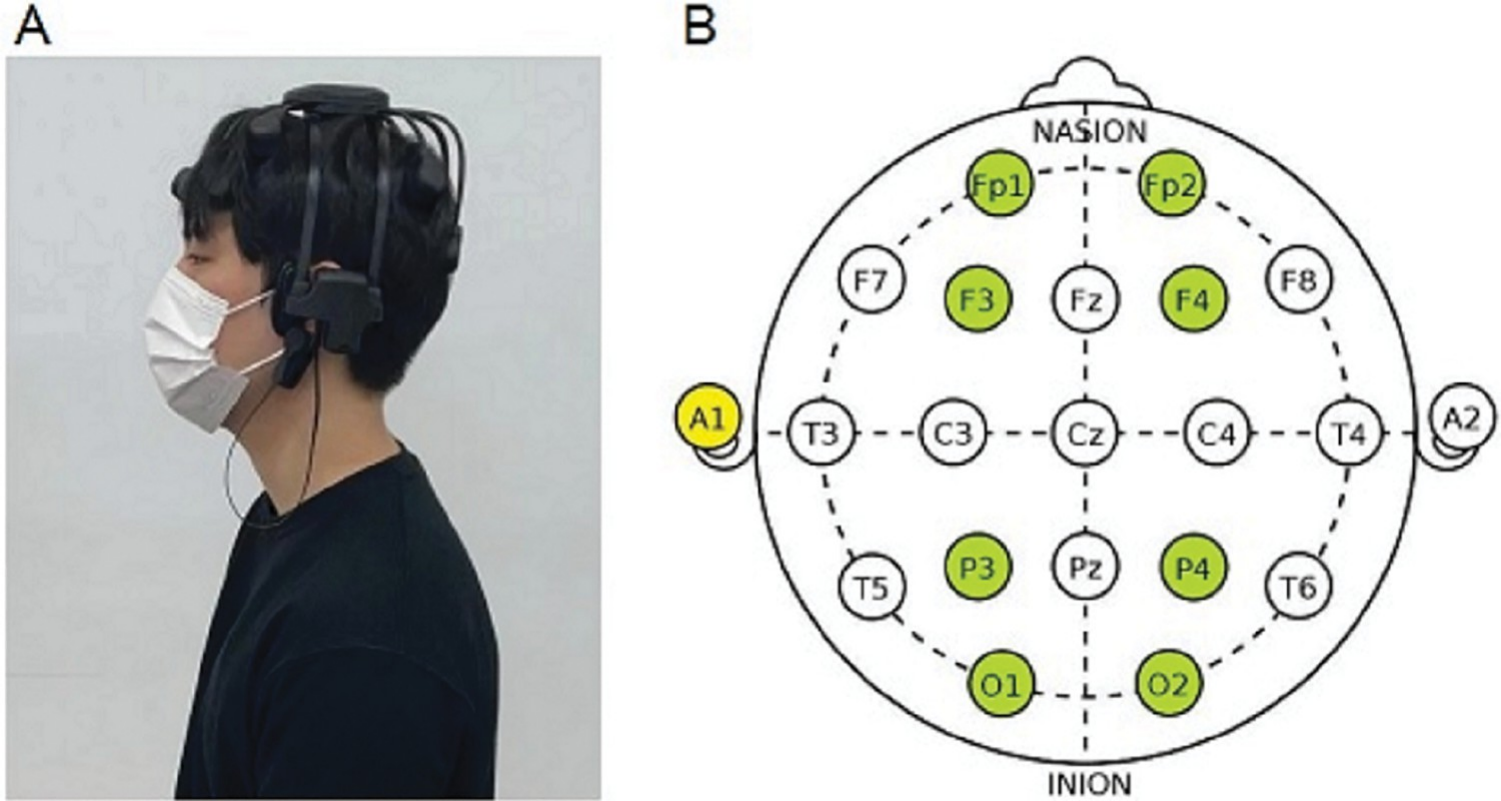
Electroencephalogram measurements. (A) Wearing the wireless dry electroencephalography device (Quick-8; Cognionics, San Diego, CA). (B) International 10–20 system of electrode placement. Highlighted sites (Fp1, Fp2, F3, F4, P3, P4, O1, and O2) indicate those measured.

EEG, a noninvasive technique, utilizes electrodes placed on the scalp to offer a precise and immediate reading of brain electrical activity [13]. We used a wireless EEG device (Quick-8; Cognionics, San Diego, CA, USA) to measure the brainwave activity of each participant during the AAAs (Fig 3A). This device consists of a dry electrode system that allows prompt removal from the scalp in case participants felt any discomfort and minimizes the hazard of electric shock compared to a wet electrode system using an electrolyte gel. Potential differences were determined by placing dry electrodes in contact with the scalp to amplify the measured electrical signals and collect data. This device is mostly used in the field of neuroscience and is certified as safe by the European Commission and Federal Communications Commission. Data were recorded using EEG measurement software (Bioteck Analysis Software, Daejeon, South Korea).

In total, eight channels were arranged to measure the brain waves: the left prefrontal lobe (Fp1), right prefrontal lobe (Fp2), left frontal lobe (F3), right frontal lobe (F4), left parietal lobe (P3), right parietal lobe (P4), left occipital lobe (O1), and right occipital lobe (O2). The electrode was attached to the left earlobe (A1) according to the International 10–20 Electrode Placement System [15] (Fig 3B).

#### 2.5.2 Subjective mood assessment

To investigate the emotional responses to each activity, the POMS, SDM, and Stress NRS were administered after the stimulation.

The POMS was developed in 2003 by McNair et al. [16]. In this study, we used the Korean version translated by Yeun and Park [17]. It consists of the following six subcategories with 30 questions: fatigue (F), depression-dejection (D), tension-anxiety (TA), anger-hostility (AH), confusion (C), and vigor (V). POMS assesses the participant’s momentary mood or feeling state, each question enquires how well each emotion describes how they feel “right now.” Responses are rated on a 5-point scale from “*not at all*” (1) to “*extremely*” (5). Total mood disturbance (TMD) is evaluated by summing the values of each subcategory (F + D + TA + AH + C–V). The lower the value, the better the participant’s positive emotional state [16, 18].

The SDM is a questionnaire to choose between adjectives and evaluates how a participant’s emotional state changes with their environment. The SDM was developed by Osgood in 1952 [19] and includes three questions: comfortable, natural, and relaxed. For each emotional state question, participants choose from 13 scoring stages (very comfortable to very uncomfortable, very natural to very artificial, and very relaxed to very awake), and the emotional state is more positive as the result value is higher.

The Stress NRS is a subjective rating scale that tracks stress levels through numerical expressions [20]. A single question is asked to measure momentary stress on a scale of 0 to 10, where 0 indicates no stress and 10 indicates the worst stress possible. Participants respond by circling the number that corresponds to their current level of stress.

### 2.6. Statistical analysis

Brain wave analysis was performed using Bioteck Analysis Software (Bioteck; Daejeon, South Korea). An EEG is a waveform that records changes in electrical signals in the brain and can be divided into different frequency bands, such as theta, alpha, beta, and gamma, which reflect different conditions, such as sensory, cognitive, and motor processes [21]. Alpha and beta waves are considered the most closely related to human emotions. This study evaluated changes in EEG signals, specifically alpha waves, which indicate stability and relaxation [22], and beta waves, which indicate attention and concentration [23, 24]. Alpha waves can be subdivided into slow and fast waves [25], and beta waves into three subregions: low, middle, and high, based on their frequency [26]. The collected raw EEG data were analyzed for relative alpha (RA), relative slow alpha (RSA), relative fast alpha (RFA), relative beta (RB), relative low beta (RLB), and relative mid beta (RMB) power spectra, as shown in Table 2.

**Table 2:** EEG Power spectrum indicators used.

Analysis indicators	Full name of the EEG power spectrum indicator	Wavelength range (Hz)
RA	Relative alpha	(8–13) / (4–50)
RSA	Relative slow alpha	(8–11) / (4–50)
RFA	Relative fast alpha	(11–13) / (4–50)
RB	Relative beta	(13–30) / (4–50)
RLB	Relative low beta	(12–15) / (4–50)
RMB	Relative mid beta	(15–20) / (4–50)

Descriptive statistical analyses were performed using SPSS version 25 for Windows (IBM Corp., Armonk, NY, USA). For the EEG and subjective evaluation data, a one-way analysis of variance (ANOVA) and Duncan’s post-hoc analysis were used to compare each activity. A *p*-value of < 0.05 was considered statistically significant. Demographic information was analyzed using Microsoft Excel (Microsoft Office 365 ProPlus; Microsoft, Redmond, WA, USA) to generate descriptive statistics of the mean, standard deviation (SD), and percentages for sex, age, height, and weight.

## 3. Results

### 3.1. Demographic characteristics

This study includes 30 adults in their 20s to 40s (average age: 27.9 ± 8.4 years), with 15 men and 15 women (men, 26.4 ± 7.1 years; women, 29.3 ± 9.2 years). Their average height and weight were 168.4 ± 8.3 cm and 66.3 ± 15.2 kg, respectively (Table 3).

**Table 3:** Demographic characteristics.

Variable	Men	Women	Total
	Mean ± SD		
% (N)	50 (15)	50 (15)	100 (30)
Age (years)	26.4 ± 7.1	29.3 ± 9.2	27.9 ± 8.4
Body Height (cm)	175.6 ± 4.7	161.1 ± 3.4	168.4 ± 8.3
Body Weight (kg)	78.6 ± 12.0	54 ± 3.9	66.3 ± 15.2

### 3.2. Electroencephalogram (EEG)

The RA power spectrum analysis showed that activity in both sides of the frontal lobes (*p* < 0.001) and left prefrontal lobe (*p* < 0.05) was significantly higher when playing with the dog. Additionally, activity in both the prefrontal lobes (*p* < 0.05, *p* < 0.01, left and right, respectively) was significantly increased during the walk. The RSA power spectrum analysis showed that the playing activity was associated with increased activation in both sides of the frontal lobes (*p* < 0.01, *p* < 0.001, left and right, respectively), while the walking activity increased activation specifically in the right prefrontal lobe (*p* < 0.05). However, no significant differences were observed in the left prefrontal lobe. The RFA power spectrum analysis showed significantly higher results during the play activity on both sides of the prefrontal and frontal lobes compared to during other activities (*p* < 0.001) (Table 4).

**Table 4:** Results of the relative alpha (RA), relative slow alpha (RSA), and relative fast alpha (RFA) power spectra via the EEG according to the AAAs.

Animal-Assisted Activity	Mean ± SD			
	**RA**			
	Fp1	Fp2	F3	F4
Meet	0.162 ± 0.024 b	0.159 ± 0.025 c	0.160 ± 0.031 c	0.154 ± 0.036 d
Play	0.182 ± 0.027 a	0.181 ± 0.026 ab	0.203 ± 0.038 a	0.199 ± 0.027 a
Feed	0.172 ± 0.027 ab	0.172 ± 0.025 abc	0.177 ± 0.042 bc	0.181 ± 0.039 ab
Massage	0.167 ± 0.022 b	0.164 ± 0.018 c	0.177 ± 0.034 bc	0.172 ± 0.037 bc
Groom	0.174 ± 0.022 ab	0.172 ± 0.025 abc	0.179 ± 0.028 bc	0.173 ± 0.026 bc
Photograph	0.166 ± 0.028 b	0.164 ± 0.026 c	0.171 ± 0.039 c	0.161 ± 0.032 cd
Hug	0.171 ± 0.032 ab	0.169 ± 0.031 bc	0.181 ± 0.042 bc	0.180 ± 0.041 b
Walk	0.184 ± 0.017 a	0.184 ± 0.022 a	0.193 ± 0.031 ab	0.187 ± 0.027 ab
**Significance**	0.013 *	0.002 **	0.000 ***	0.000 ***
	**RSA**			
	Fp1	Fp2	F3	F4
Meet	0.114 ± 0.020	0.112 ± 0.019 bc	0.106 ± 0.022 c	0.101 ± 0.027 c
Play	0.118 ± 0.017	0.119 ± 0.021 ab	0.132 ± 0.029 a	0.130 ± 0.022 a
Feed	0.117 ± 0.019	0.117 ± 0.018 abc	0.117 ± 0.029 bc	0.119 ± 0.026 ab
Massage	0.112 ± 0.017	0.108 ± 0.015 c	0.115 ± 0.024 bc	0.112 ± 0.026 bc
Groom	0.117 ± 0.015	0.115 ± 0.016 abc	0.117 ± 0.022 bc	0.114 ± 0.019 bc
Photograph	0.112 ± 0.021	0.111 ± 0.020 bc	0.112 ± 0.028 c	0.103 ± 0.022 c
Hug	0.116 ± 0.023	0.114 ± 0.023 bc	0.118 ± 0.032 abc	0.118 ± 0.03 ab
Walk	0.124 ± 0.013	0.125 ± 0.015 a	0.129 ± 0.023 ab	0.124 ± 0.02 ab
**Significance**	0.217 ^NS^	0.017 *	0.004 **	0.000 ***
	**RFA**			
	Fp1	Fp2	F3	F4
Meet	0.048 ± 0.006 c	0.048 ± 0.007 c	0.055 ± 0.012 c	0.053 ± 0.011 c
Play	0.064 ± 0.013 a	0.062 ± 0.008 a	0.071 ± 0.012 a	0.069 ± 0.008 a
Feed	0.056 ± 0.009 b	0.055 ± 0.009 b	0.060 ± 0.015 bc	0.062 ± 0.015 b
Massage	0.054 ± 0.008 b	0.056 ± 0.009 b	0.061 ± 0.010 b	0.061 ± 0.012 b
Groom	0.057 ± 0.009 b	0.057 ± 0.011 b	0.062 ± 0.008 b	0.059 ± 0.009 b
Photograph	0.054 ± 0.009 b	0.054 ± 0.008 b	0.059 ± 0.012 bc	0.058 ± 0.013 bc
Hug	0.054 ± 0.011 b	0.055 ± 0.012 b	0.062 ± 0.013 b	0.062 ± 0.014 b
Walk	0.059 ± 0.006 ab	0.059 ± 0.008 ab	0.065 ± 0.010 b	0.063 ± 0.009 b
**Significance**	0.000 ***	0.000 ***	0.000 ***	0.000 ***

Note: Post-hoc analysis: a > b > c > d via Duncan’s multiple range test; ^6 NS^ = non-significant; *, **, *** = significant at *p* < 0.05, 0.01, and 0.001 via ANOVA; SD = standard deviation.

The RB power spectrum analysis showed significantly higher activity during the play activity in both the prefrontal lobes (*p* < 0.01, *p* < 0.001, left and right, respectively), whereas the massage activity was associated with increased activation in the right frontal lobe (*p* < 0.05). The RLB power spectrum analysis showed significantly higher activity during massage and grooming activities on both sides of the prefrontal lobes and the left frontal lobe (*p* < 0.001). And the massage activity was also associated with increased activation in the right frontal lobe (*p* < 0.001). Furthermore, the RLB index showed significantly increased brain activity in all parts of the parietal and occipital lobes during the massage and grooming activities (*p* < 0.001). However, no significant differences were found in the parietal and occipital lobes in the other power spectra for any activity. The RMB power spectrum analysis showed that the playing activity was associated with increased activation in both sides of the prefrontal lobes (*p* < 0.001) and left frontal lobe (*p* < 0.05). However, no significant differences were observed in the participants’ right frontal lobe (Table 5).

**Table 5:** Results of the relative beta (RB), relative low beta (RLB), and relative mid beta (RMB) power spectra via the EEG according to the AAAs.

Animal-Assisted Activity	Mean ± SD			
	**RB**			
	Fp1	Fp2	F3	F4
Meet	0.253 ± 0.056 c	0.252 ± 0.043 c	0.314 ± 0.035	0.320 ± 0.030 abc
Play	0.294 ± 0.031 a	0.299 ± 0.030 a	0.315 ± 0.036	0.305 ± 0.035 bc
Feed	0.281 ± 0.038 ab	0.276 ± 0.039 b	0.314 ± 0.040	0.308 ± 0.031 bc
Massage	0.288 ± 0.037 ab	0.288 ± 0.032 ab	0.322 ± 0.021	0.328 ± 0.028 a
Groom	0.290 ± 0.036 ab	0.289 ± 0.033 ab	0.319 ± 0.043	0.312 ± 0.027 abc
Photograph	0.284 ± 0.035 ab	0.280 ± 0.031 ab	0.321 ± 0.034	0.323 ± 0.034 ab
Hug	0.270 ± 0.043 bc	0.275 ± 0.051 b	0.313 ± 0.044	0.306 ± 0.041 bc
Walk	0.273 ± 0.042 abc	0.271 ± 0.038 b	0.308 ± 0.036	0.304 ± 0.027 c
**Significance**	0.003 **	0.000 ***	0.858 ^NS^	0.017 *
	**RLB**			
	Fp1	Fp2	F3	F4
Meet	0.062 ± 0.009 d	0.060 ± 0.008 d	0.071 ± 0.012 d	0.070 ± 0.011 d
Play	0.082 ± 0.010 b	0.084 ± 0.011 b	0.094 ± 0.012 b	0.088 ± 0.008 c
Feed	0.073 ± 0.013 bcd	0.072 ± 0.011 c	0.080 ± 0.017 cd	0.080 ± 0.014 c
Massage	0.288 ± 0.037 a	0.288 ± 0.032 a	0.322 ± 0.021 a	0.328 ± 0.028 a
Groom	0.290 ± 0.036 a	0.289 ± 0.033 a	0.319 ± 0.043 a	0.312 ± 0.027 b
Photograph	0.072 ± 0.010 bcd	0.072 ± 0.009 c	0.079 ± 0.014 cd	0.079 ± 0.013 c
Hug	0.070 ± 0.014 cd	0.071 ± 0.015 c	0.079 ± 0.015 cd	0.079 ± 0.013 c
Walk	0.075 ± 0.009 bc	0.073 ± 0.009 c	0.085 ± 0.013 bc	0.081 ± 0.009 c
**Significance**	0.000 ***	0.000 ***	0.000 ***	0.000 ***
	**RLB**			
	P3	P4	O1	O2
Meet	0.081 ± 0.012 b	0.081 ± 0.017 b	0.081 ± 0.017 b	0.080 ± 0.016 b
Play	0.085 ± 0.010 b	0.085 ± 0.015 b	0.086 ± 0.008 b	0.086 ± 0.012 b
Feed	0.081 ± 0.019 b	0.079 ± 0.015 b	0.080 ± 0.011 b	0.080 ± 0.010 b
Massage	0.349 ± 0.022 a	0.349 ± 0.033 a	0.342 ± 0.036 a	0.346 ± 0.039 a
Groom	0.341 ± 0.027 a	0.347 ± 0.036 a	0.346 ± 0.037 a	0.347 ± 0.033 a
Photograph	0.079 ± 0.013 b	0.080 ± 0.013 b	0.079 ± 0.010 b	0.081 ± 0.012 b
Hug	0.082 ± 0.012 b	0.082 ± 0.012 b	0.081 ± 0.009 b	0.083 ± 0.009 b
Walk	0.081 ± 0.008 b	0.080 ± 0.010 b	0.084 ± 0.009 b	0.083 ± 0.009 b
**Significance**	0.000 ***	0.000 ***	0.000 ***	0.000 ***
	**RMB**			
	Fp1	Fp2	F3	F4
Meet	0.078 ± 0.018 c	0.078 ± 0.012 c	0.099 ± 0.011 c	0.100 ± 0.009
Play	0.100 ± 0.013 a	0.101 ± 0.012 a	0.110 ± 0.011 a	0.106 ± 0.013
Feed	0.091 ± 0.014 b	0.089 ± 0.012 b	0.101 ± 0.014 bc	0.100 ± 0.008
Massage	0.093 ± 0.013 ab	0.091 ± 0.011 b	0.106 ± 0.008 abc	0.106 ± 0.010
Groom	0.093 ± 0.014 ab	0.093 ± 0.013 b	0.105 ± 0.014 abc	0.101 ± 0.008
Photograph	0.092 ± 0.012 ab	0.090 ± 0.009 b	0.103 ± 0.011 abc	0.103 ± 0.010
Hug	0.087 ± 0.015 b	0.090 ± 0.020 b	0.102 ± 0.016 bc	0.099 ± 0.013
Walk	0.092 ± 0.016 ab	0.092 ± 0.015 b	0.106 ± 0.014 ab	0.105 ± 0.012
**Significance**	0.000 ***	0.000 ***	0.023 *	0.071 ^NS^

Note: Post-hoc analysis: a > b > c > d via Duncan’s multiple range test; ^NS^ = non-significant; *, **, *** = significant at *p* < 0.05, 0.01, and 0.001, respectively, via ANOVA; SD = standard deviation.

### 3.3. Subjective evaluations of the emotional states

**Fig 4:**
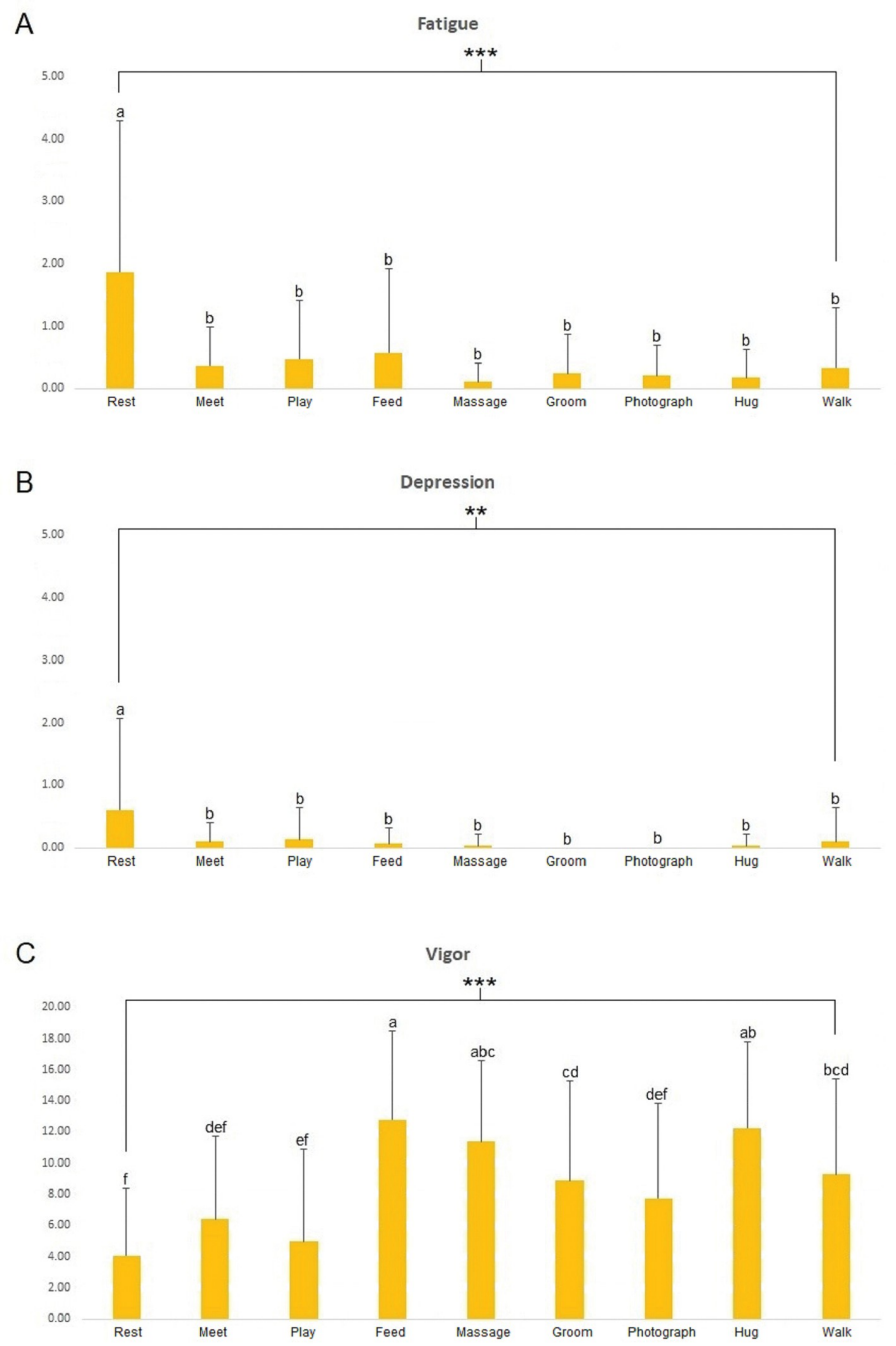
Comparisons of fatigue (A), depression-dejection (B), vigor (C) scores in the POMS for each activity. **, *** = significant at *p* < 0.01 and 0.001 via ANOVA. Post-hoc analysis: a > b > c > d >e >f via Duncan’s multiple range test.

**Fig 5:**
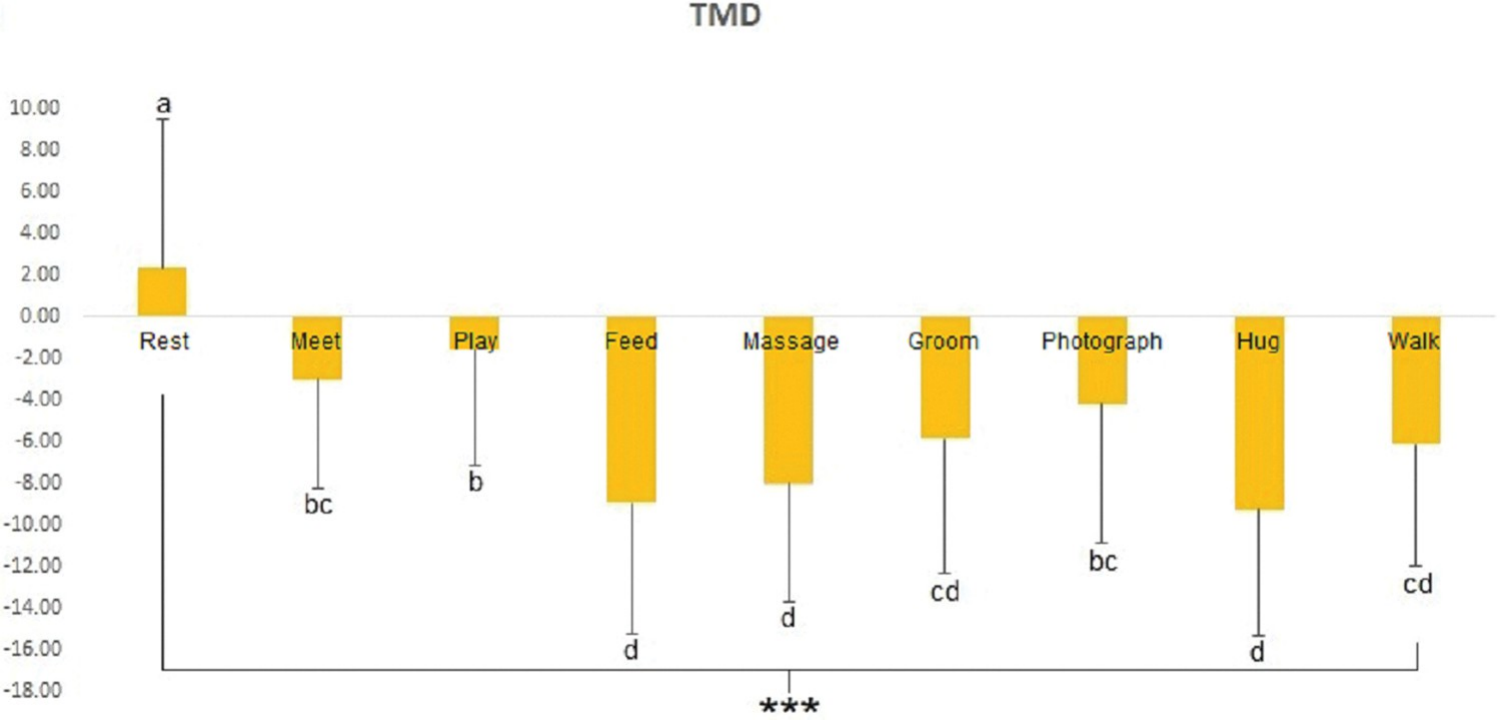
Comparisons of total mood disturbance (TMD) scores in the POMS for each activity. *** = significant at *p* < 0.001 via ANOVA. Post-hoc analysis: a > b > c > d via Duncan’s multiple range test.

The POMS evaluated mood states subjectively in accordance with AAAs. The results were divided into six subcategories for analysis (fatigue, depression-dejection, tension-anxiety, anger-hostility, confusion, and vigor). Participants showed significantly lower fatigue (*p* < 0.001) (Fig 4A) and depression (*p* < 0.01) (Fig 4B) in all AAAs than at rest. Furthermore, participants experienced the most vigorous mood states when feeding the dog (*p* < 0.001) (Fig 4C). No significant differences in tension-anxiety, anger-hostility, or confusion were observed. The sum of the six categories and analysis of the TMD revealed significantly lower TMD values when participants were feeding, massaging, and hugging the dog, which indicated that they had positive mood states (*p* < 0.001) (Fig 5).

**Fig 6:**
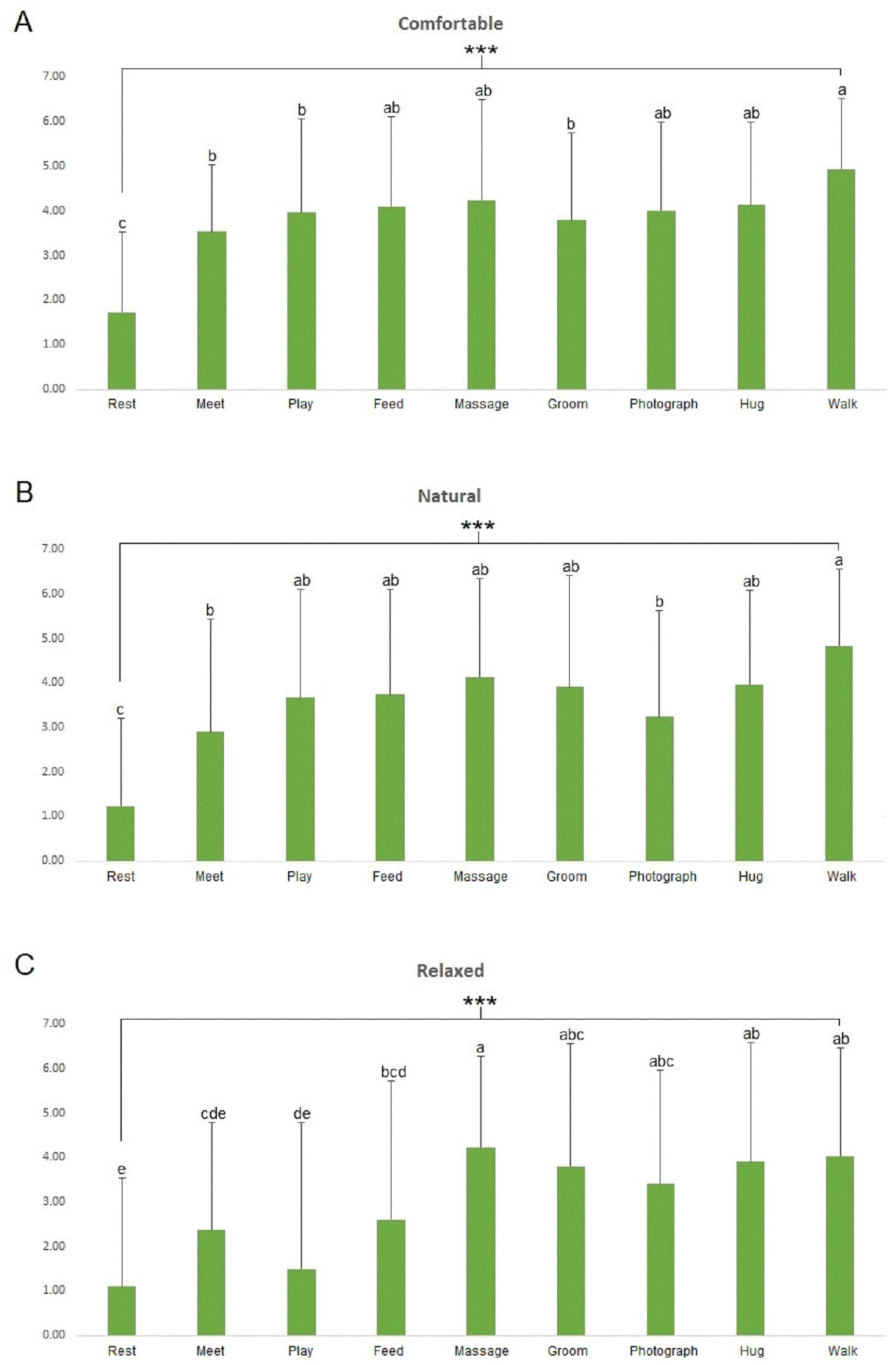
Comparisons of comfortable (A), natural (B), and relaxed (C) scores in the SDM for each activity. *** = significant at *p* < 0.001 via ANOVA. Post-hoc analysis: a > b > c > d > e via Duncan’s multiple range test.

Results of the SDM revealed that walking with the dog showed significantly higher “comfortable” (*p* < 0.001) and “natural” (*p* < 0.001) feelings compared to the other activities. Furthermore, participants felt significantly more “relaxed” (*p* < 0.001) when they performed the massage activity (Fig 6).

**Fig 7:**
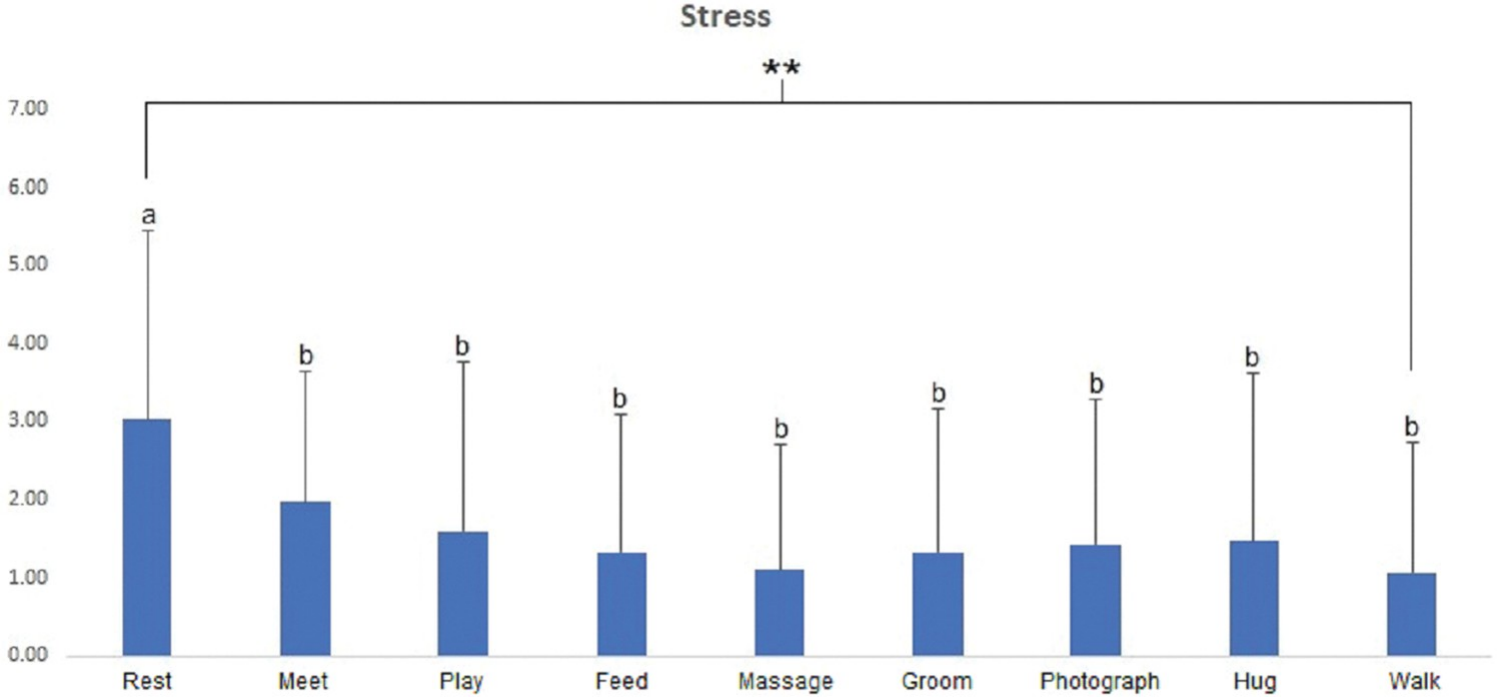
Comparisons of the Stress NRS scores for each activity. ** = significant at *p* < 0.01 via ANOVA. Post-hoc analysis: a > b via Duncan’s multiple range test.

In the Stress NRS, participants showed significantly lower stress in all AAAs than at rest (*p* < 0.01) (Fig 7).

## 4. Discussion

This study investigated the psychophysiological and emotional responses to AAI by activity type. The EEG records of healthy adults during diverse activities with a dog were analyzed. Results revealed that the activity type elicited different brainwave reactions.

Playing and walking with a dog increased brain activity in the RA and RSA power spectrum indices of the prefrontal and frontal lobes. Previous studies have reported that increased alpha power reflects relaxation and emotional stability [22] and is associated with improved memory and reduced mental stress [27, 28]. The RSA wave generally appears when the body is relaxed and in a meditative state [29, 30]. Playing with the dog also increases brain activity in the RFA index of the prefrontal and frontal lobes. The RFA signifies an attention concentration in a relaxed condition [29, 31]. Increased RFA indicates that the brain is awake in a stable state for mental rotation tasks and correlates with cognitive judgment, learning ability, and creative thinking [32]. Hence, these findings can serve as a basis for the development of AAI programs for emotional relaxation and stress management.

The frontal lobe, the most important functional area of the brain, is divided into the motor and prefrontal lobes. The frontal lobe is involved in the control of physical movements and is responsible for moral behavior and various cognitive functions, such as problem-solving, language, and attention [33, 34]. The prefrontal cortex is the anterior part of the frontal lobe and receives input from all other cortical regions and intelligently controls our thoughts, behaviors, movement, and emotions through extensive connections [35, 36]. The prefrontal lobe plays a role in the regulation of complex cognitive, memory, emotional, language, and behavioral functioning. Furthermore, it is associated with various higher cognitive functions, such as task memory, attention focus, abstract reasoning, social interaction, goal-oriented behavior, and problem solving [37–39]. Activation of the frontal and prefrontal lobes is related to cognitive function, which indicates an improvement in intellect and attention.

Therefore, based on our results, we assumed that an increase in the RA, RSA, and RFA power in the prefrontal and frontal lobes during walking and playing with the dog implies improvement in relaxation and emotional stability. This result is in accordance with a previous study on horseback riding exercise therapy, which reported that the RSA and RFA power activated after the horseback riding program compared with the control group [31, 40]. Many previous studies have reported that interactions with dogs in AAA settings increase oxytocin levels [41–43]. Furthermore, friendly interactions with dogs were linked to decreased cortisol levels [41, 44]. Hormonal indicators, such as cortisol and oxytocin levels, could objectively reflect stress-related physiological responses of the endocrine and autonomic nervous systems. Mental processes influence bodily physiology and, in turn, influence thoughts and feelings [45]. This is related to the results of our subjective evaluations of emotional states. The results of the SDM showed that participants felt significantly "comfortable" and "natural" when walking with the dog. Furthermore, the results of the Stress NRS showed that participants reported significantly lower stress levels in all activities involving a dog. An increase in the alpha power spectrum in the prefrontal and frontal lobes supports the evidence that human-dog interactions decrease stress and can lead to emotional stability.

In the RB power spectrum index of the prefrontal and frontal lobes, brain activity increased while playing with and massaging the dog. In previous studies, an increase in the beta power band indicated that the brain was alert, focused [23], and attentive [24], and had motor functions [46]. The brain activity of the RMB power spectrum index of the prefrontal and frontal lobes increased while playing with the dog. RMB power spectra appeared during concentration [47], problem-solving, logical thinking, and interest in external objects [48]. In particular, the RLB power spectrum index showed significantly higher brain activity during massage and grooming in all eight channels of the prefrontal, frontal, parietal, and occipital lobes.

The parietal lobe, which is positioned between the frontal and occipital lobes, plays a vital role in integrating sensory information from different body parts, perceiving stimuli, comprehending spatial orientation, and controlling motor functions [34, 49]. The occipital region is located posteriorly in the human cerebral cortex and is responsible for processing visual information [50]. The occipital lobe is normally activated upon visual stimulation and is a center for visual information primarily responsible for receiving and transmitting visual information [51]. Activation in the parietal and occipital lobes during massage and grooming activities suggests that the participants focus on the dog’s body to perform the activities correctly. The RLB frequency band was observed in the sensory-motor cortex of the brain and is also known as the sensorimotor rhythm (SMR) [52], involved in cognitive functions, such as reaction time, psychomotor skills, and spatial ability [53, 54]. The RLB is primarily activated during a state of relaxed concentration while maintaining a stress-free condition [48, 55]. Therefore, these activities can be referred to as the AAI program for participants who expect the positive health effects of increased concentration.

An increase in RB, RLB, and RMB indices in the activities of playing, massaging, and grooming the dog may help improve or maintain attention and concentration without stress. Our results corresponded with those of previous studies on AAT after pediatric surgery, in which children who had sessions with a therapy dog showed faster EEG beta activity [56]. In another study, participants showed higher frontal lobe brain activity when they interacted with a dog compared to a stuffed animal. In addition, frontal lobe brain activity increased as the intensity of contact with the animal increased [57]. Our study showed similar results. Brain activity in the RLB power index, such as massage and grooming activities, increased as the intensity of contact with the animal increased. This is connected to the results obtained from our subjective evaluations of emotional states. Evaluations of the POMS revealed that participants experienced increased positive mood states during activities such as massage, feeding, and hugging. The results of the SDM also showed that participants reported feeling significantly more "relaxed" when they engaged in the massage activity. Based on these results, it will be possible to develop an AAI program that increases positive health effects from increased concentration.

This study includes participants who both have pets and those who do not have any. However, individuals who primarily participate in animal-assisted activities are those that enjoy being around animals and do not have any associated fears. It is important to consider a limitation where individuals with prior experience or a fondness for animals may respond differently to animal-assisted activities, which could potentially introduce bias into the study results. Another possible limitation of this study was the small sample size. Studies correlating brain activity mechanisms to the effects of human-animal interaction are still in the early stages and lack sufficient data. It is crucial to conduct further studies with a larger number of participants to confirm the positive role of AAI on brain activity.

## 5. Conclusion

Our study demonstrates that animal interaction activities, such as playing, walking, massaging, and grooming dogs, have a positive effect by facilitating increased brain activity in healthy participants. This indicates that certain activities activate relaxation, emotional stability, attention, concentration, and creativity. Notably, playing with the dog has an affirmative effect on both relaxation and concentration. Additionally, through a subjective mood assessment, results revealed that interactions with dogs can decrease human stress and induce positive emotional responses.

These results provide data that form the basis for the composition of the AAI program. They may be applicable as a reference to determine the most effective activities for specific participant categories in AAI. In future studies, confirming the validity of these findings and elucidating the correlation between specific activities and brain wave patterns will be necessary to better understand the mechanisms behind the effects of human-animal interaction.
